# Quality of Life in Female Breast Cancer Patients and Survivors in a South African Municipality

**DOI:** 10.1177/11782234241282519

**Published:** 2024-10-08

**Authors:** Rebecca Wilkinson, Lynn Smith

**Affiliations:** Department of Sport and Movement Studies, Faculty of Health Sciences, University of Johannesburg, Johannesburg, South Africa

**Keywords:** Breast cancer, quality of life, cancer screening, cancer survival, remission

## Abstract

**Background:**

Breast cancer diagnosis and treatment processes affect patients physically and mentally, and have an impact on their quality of life, even years after receiving treatment.

**Objectives:**

The objective of this study was to determine the quality of life in female breast cancer patients and survivors in a South African context. The municipality within which participants were recruited for this study was Ekurhuleni, based in the Gauteng province, South Africa.

**Design:**

This study followed a cross-sectional research design. Quantitative data was collected.

**Methods:**

The Quality-of-Life Patient/Cancer Survivor Version (2012) was used to determine participants’ quality of life in 4 subscales, namely, physical, psychological, social, and spiritual. The questionnaire was accessible to participants via the online Google Forms platform as well as in hard-copy format at local medical facilities. The Statistical Package for Social Sciences (SPSS) was used to compute statistics, and the level of significance was set at 95% (*P* < .05).

**Results:**

One hundred female breast cancer patients and survivors from the region of Ekurhuleni, South Africa, took part in this study. The findings demonstrate that the quality-of-life subscale with the highest score was spiritual well-being (6.66 ± 2.07) and the lowest was psychological well-being (4.91 ± 1.93). No significant difference was found between quality of life and type of facility attended. Significant differences were found in quality-of-life ratings between breast cancer patient and breast cancer survivor populations.

**Conclusion:**

Breast cancer can result in a compromised quality of life, and with the increased prevalence and survival rate of breast cancer patients, both the short- and long-term effects of the condition and its treatments are heightened.

## Introduction

The most recent municipal cancer data for South Africa was collected in 2018 in Ekurhuleni, Gauteng, which saw breast cancer contributing to 12% of all cancer cases in the region, with 553 females diagnosed with this disease.^1^ Improvements in breast cancer screening, diagnosis, and treatment have resulted in a significant increase in the size of the breast cancer survivor population.^2^ However, breast cancer patients and survivors may face unique challenges in their physical and mental well-being due to their diagnosis and associated treatments.^3^

Quality of life relates to the subjective perception of physical, mental, and external factors and is an important factor in both breast cancer patients and survivors.^4^ One’s quality of life, measured by the various subscales, such as the psychological, physical, social, and spiritual, can be negatively affected by breast cancer, with research suggesting these effects may be long term.^5^

The value of measuring quality of life has increasingly been recognised.^6^ Nonetheless, quality of life in the breast cancer population may be overlooked as the primary focus is on treating cancer and achieving a state of remission, rather than on the person’s holistic well-being during this period.^7^ There has been a shift in focusing on how well cancer patients are surviving compared with simply how long they are surviving.^8^

## Methods

### Study design

Quantitative data was collected through a close-ended questionnaire. The study was cross-sectional in design.

### Selection and recruitment

Those who met the inclusion criteria were invited to participate in the study. Participation involved completing the questionnaire, either online through the Google Forms platform or a hard copy. The questionnaire link and hard-copy questionnaires were made available to qualifying participants at various health care facilities and practices as well as online on social media platforms and breast cancer support groups.

Inclusion criteria include the following:

Female sex.
Clinically diagnosed with breast cancer who were either undergoing treatment for breast cancer and those who had survived breast cancer.
Residents of the Ekurhuleni municipality of Gauteng, South Africa, at the time of data collection.
Internet access for completion of the online questionnaire.

### Questionnaires

The initial questions were related to basic demographics and breast cancer diagnosis of the participant. In the demographics section, no personal information was gathered, therefore making participation anonymous. The Quality-of-Life Patient/Cancer Survivor Version (QOL-CSV) was used to investigate quality-of-life ratings (Ferrell, Hassey-Dow, and Grant 2012). The QOL-CSV has a reliability of 0.89 and 0.93, and a validity of 0.78.

### Statistical analysis

Descriptive and inferential statistics were computed in this study. Statistical analysis was completed using the Statistical Package for Social Sciences (SPSS, Version 28), which included descriptives, comparisons, and differences. The normality of the data was assessed by making use of the Kolmogorov-Smirnov and Shapiro-Wilk tests. Linear correlations were computed to determine whether any differences existed between quality of life between type of facility attended for care (private compared with public) and breast cancer status (patient compared with survivor). These differences were calculated using *t*-tests and 2-sided *P*-value as the normality of the data was established. The level of significance was set at 95% (*P* < .05).

### Research ethics

Prior to data collection, this study was approved by the Institutions Research Ethics committee (REC-1048-2021). Gatekeeper permission was sought and granted by each of the health care facilities and practitioners where data was collected. Informed consent was obtained by each participant.

## Results

### Demographics

The mean age of the participants in this study was 56 years, with 27 years and 85 years being the youngest and oldest ages of the participants, respectively. The mean age of breast cancer diagnosis was 50 years. The youngest age of diagnosis was 24 years and the oldest was 84 years. The 2 participants who reported a reoccurrence in their breast cancer had 4 (2003 to 2007) and 26 (1994 to 2020) years between their initial and reoccurring diagnoses, respectively. For these 2 participants, their most recent age and year of diagnosis was captured and used for the purpose of this research. On further analysis, the sample was split into breast cancer patients and breast cancer survivors. The mean age of the breast cancer patient group was 54 years with a mean diagnosis age of 52 years. The breast cancer survivor group had a mean age of 57 years and a mean age at diagnosis of 50 years. The most commonly reported year of breast cancer diagnosis among the participants was 2019 (16%).

The participants were required to select the type of facility they attended for their breast cancer diagnosis and treatments. Most of this sample (79%) attended private facilities.

**Figure 1.**
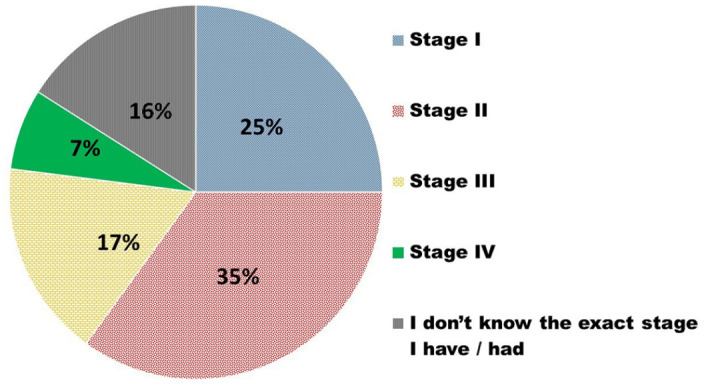
Stage of breast cancer.

In terms of type of breast cancer, the most selected response was that the participants were not sure of the type of breast cancer they had (29%). The most and least commonly selected types of breast cancer were invasive ductal/lobular carcinoma in situ (22%) and locally advanced breast cancer (2%), respectively. For those who selected ‘other’, the specified responses included oestrogen positive (5%), HER positive (2%), squamous adenocarcinoma (1%), and triple negative (6%). As seen in Figure 1, the most common stage of breast cancer among the participants in this study was stage II, with 16% of the participants not knowing the stage of their breast cancer diagnosis.

As seen in Table 1, the participants were required to select which treatment modalities they had received for their breast cancer. Two percent of participants elected not to have treatment but had selected that they had surgery for their breast cancer. In terms of remission status, 67% of the participants reported they were currently in remission for their breast cancer. One percent reported that they were not in remission but were no longer receiving treatment. Of those who were in remission, the reported period of remission was 14.9%, 38.8%, and 46.3% for less than 1 year, 1 to 5 years, and more than 5 years, respectively. Of the 100 participants in this study, 42% were undergoing breast cancer treatments at the time of participation.

**Table 1.** Treatment modalities received by the participants.

Type of treatment	Percentage (%)
Surgery/operation	82%
Chemotherapy	60%
Radiation therapy	44%
Hormone therapy	38%
Decided not to have treatment	2%
Other (specified)	13%

The participants were required to declare comorbidities with which they were diagnosed in addition to breast cancer, the results of which are seen in Table 2. Forty-two percent of participants reported no comorbidities. The most reported comorbidity was hypertension, which was reported by 27% of the participants. Notably, 2% of participants reported having comorbidities but did not specify which one(s).

**Table 2.** Reported comorbidities of the participants.

Comorbidity	Percentage (%)
Hypertension/high blood pressure	27
Diabetes/pre-diabetes/insulin resistance	9
Arthritis	9
Dyslipidemia/high cholesterol	7
Hypothyroidism/under-active thyroid	6
Anxiety	2
AV malformation	2
Melanoma/skin cancer	2
Blood clots	2
Depression	2
Glanzmann thrombasthenia	2
Kasabach-Meritt syndrome	2
Lymphedema	2
Osteopenia	2
Osteoporosis	2
Angiosarcoma	1
Autoimmune disease	1
Diverticulitis	1
Fibromyalgia	1
Goitre	1
Human papillomavirus	1
Lung cancer	1
Lichen sclerosis	1
Parkinson’s disease	1
Rheumatoid arthritis	1
Spinal degeneration	1

### Quality of life

**Figure 2.**
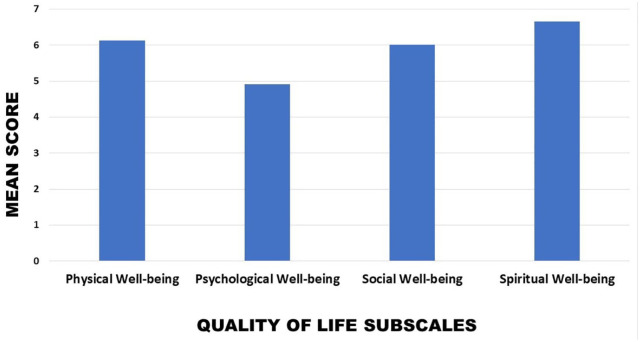
Mean quality-of-life scores in the 4 subscales.

The mean quality-of-life scores is displayed in Figure 2. The lowest mean quality of life was seen for the psychological well-being subscale (4.91 ± 1.93), and the highest score was observed in the spiritual well-being subscale (6.66 ± 2.07).

### Comparisons

**Figure 3.**
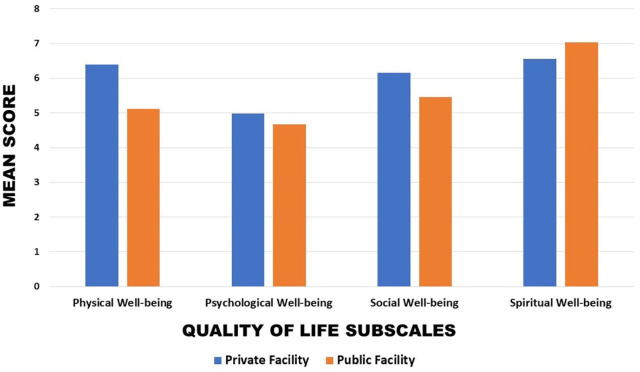
Mean quality-of-life scores in those attending private facilities and public facilities.

On further analysis, as illustrated in Figure 3, it was seen that the mean physical, psychological, and social quality of life was reportedly higher among those attending private facilities when compared with those attending public facilities. In contrast to the 3 other quality-of-life subscales, a higher mean for spiritual well-being was seen in those attending public facilities. However, this result should be interpreted with caution due to the large difference in the sizes of groups.

**Figure 4.**
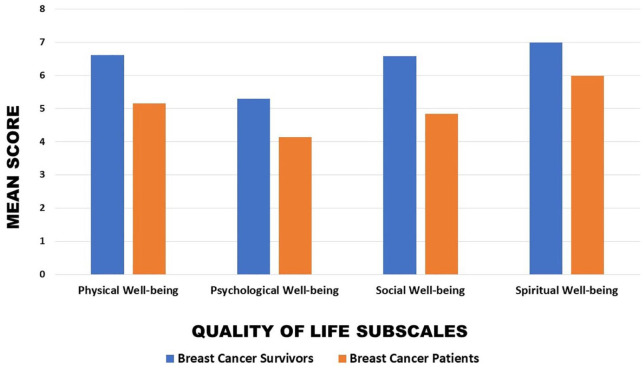
Mean quality-of-life scores in those classified as breast cancer survivors and breast cancer patients.

When comparing the quality-of-life subscales between the breast cancer patients and breast cancer survivor populations, all subscales of quality of life were higher in the breast cancer survivor population (Figure 4). The differences in means between the 2 populations for physical, psychological, social, and spiritual well-being were 1.45, 1.15, 1.74, and 1.00, respectively.

## Discussion

### Demographics

The study involved breast cancer patients and survivors residing in the Ekurhuleni municipality of Gauteng, South Africa. This municipality has published some of the more recent area-specific cancer statistics in the country, which revealed that in 2018, 15 men and 553 women in the region were diagnosed with breast cancer.^1^

The mean age of participants of this study was 56 years, with 64% of participants being 59 years or younger. The risk of cancer dramatically increases with increased age, due to accumulated risk factors as well as cellular repair mechanisms becoming less effective with age.^9^ The mean age at breast cancer diagnosis among the participants was 50 years, and 50% of participants reported being diagnosed before the age of 50 years. In breast cancer cases in African women, 46.2% of cases and 39.2% of deaths occurred in women under the age of 50 years, which is consistent with research demonstrating young age profiles of breast cancer patients in Africa.^10^

Forty-two percent of participants reported having non-invasive or invasive ductal or lobular carcinoma in situ, with research demonstrating that 85% of breast cancer occurs in the breast ducts, with 15% seen in the lobules.^11^ Metastatic breast cancer, which is breast cancer that originated in the breast tissue and has spread to additional tissue in the body, has a poorer prognosis.^11^ In this sample, 8% of participants reported being diagnosed with metastatic breast cancer; however, no follow-up question regarding site of metastases was asked, which would have provided further value to the study. Notably, there were 2 participants who reported a reoccurrence of their breast cancer (ie, 2 diagnoses). Breast cancer can reoccur, potentially in 3 regions: local, regional, and distant, with research demonstrating that at least one-half of breast cancer reoccurrences occur more than 5 years after the initial diagnosis.^11,12^ The 2 participants who reported a reoccurrence in their breast cancer had 4 and 26 years between their initial and reoccurring diagnosis, respectively.

Potentially related to false perceptions that breast cancer occurs in older women, younger women are more likely to be diagnosed with a later stage of breast cancer.^13^ The most commonly reported stage of breast cancer diagnosis among these participants was stage II (35%) and the least common was stage IV (7%). In contrast to this study, in sub-Saharan Africa, it was revealed that 77% of cancers are at stage III or IV at the time of diagnosis.^14^ In a South African context, a 30% rate of late-stage presentation of breast cancer cases has previously been found, with this study revealing that 24% of cases were stage III and IV diagnoses.^14^

In this study, it was seen that 16% and 29% of participants did not know their stage of diagnosis or type of breast cancer, respectively. Another study revealed that among black South African women with breast cancer, only 1 in 50 reported that their health care provider had spoken to them about the details of their cancer diagnosis and prognosis.^15^ Some studies demonstrated that the patient’s awareness of their cancer status increased the rates of anxiety and depression, but in other studies the opposite is seen.^16^ Indeed, in Wan et al,^16^ it was found that informing patients of their diagnosis may not detrimentally affect their quality of life when compared with being uninformed.

There are various treatment modalities for breast cancer, and the modality is chosen based on location, type, stage, and grade of the cancer, as well as the patient’s general health status.^4,17^ Eighty-two percent of the participants selected surgery as a treatment modality, which is consistent with research by Lambert et al.^15^ Surgery can lead to impairments for breast cancer patients, with mastectomies being associated with a reduced quality of life,^18,19^ which has contributed to the establishment of conservative surgery or immediate reconstructive surgery as well as the use of adjuvant therapies.^18,19^ Enhanced outcomes have been seen through the use of adjuvant therapy; however, it has been known to give rise to unfavourable side effects.^20^ The second and third most reported modalities of treatment in this study were chemotherapy (60%) and radiation therapy (44%).

After 9 years of breast cancer survivorship, this population has been found to be more likely to die of heart disease than breast cancer.^2^ The most reported comorbidity in this study was hypertension, seen in 27% of the participants, which may elevate an already notable risk as breast cancer patients and survivors often possess an elevated cardiovascular, renal, and metabolic disease risk after their cancer and associated treatments.^3^ Nine participants reported each of arthritis and diabetes or insulin resistance as comorbidities. Cancer survivors do appear to have an elevated type 2 diabetes mellitus risk compared with the general population.^21^ It is not only the toxicity of cancer treatments that place survivors at risk for developing comorbidities, but also their lifestyle habits.^21^ Due to the elevated risk of comorbidities after cancer, because of either the cancer itself or the associated treatments, directing focus to secondary prevention strategies is becoming a priority and may offer a more cost-effective solution to maintaining the survivor’s health.^21^ The researcher does acknowledge that as comorbidity status was self-reported, this may not be an accurate representation of the comorbidity presence in the population. Future studies should potentially access medical records to confirm comorbidity diagnoses.

### Quality of life

A woman’s breast cancer experience is individualised but may include phases, such as diagnosis, treatment, side effects of treatments, completing treatment and re-entering normal living, survivorship, reoccurrence, and palliative care, all of which have an effect on quality of life.^22^ Research has shown that assessing the cancer patient’s quality of life could aid in improving treatment and serve as a prognostic factor.^5^ As women are now more likely to experience earlier breast cancer diagnosis and have increased life expectancy, it is crucial to consider the effects of breast cancer and its treatments during and after their cancer journey.^5^ By making use of the quality-of-life patient/cancer survivor questionnaire, this study investigated quality of life in 4 subscales, namely, physical, psychological, social, and spiritual.

When grouping the questions into the 4 subscales, it was seen that, in terms of overall rating, the lowest mean quality-of-life subscale was psychological well-being (4.9) and the highest the subscale spiritual well-being (6.66). Interestingly, and in contrast to this study, in Imran et al,^19^ the highest quality-of-life score was for social functioning and lowest for physical functioning. The different subscales measuring quality of life represent a highly interrelated and dynamic system, and although we separate the different aspects into various subscales, the subscales influence each other.^7^

Regarding quality of life, when reviewing the participants’ ratings, one must consider that quality of life is individual-centred and self-reported with factors such as age, disease severity and status, phase of cancer continuum, treatment modalities, and side effects impacting an individual’s quality of life.^7,19^ It is also important to consider that internal and external causes, other than breast cancer, can have an effect on quality of life, including physical and psychological comorbidities, health care delivery and accessibility, socioeconomic factors, and personal relationships.^7^ As such, a holistic approach incorporating quality of life and lifestyle is important in breast cancer treatment and recovery.^22^

### Differences in quality of life and physical activity levels exist between type of facility attended and breast cancer status

#### Public and private facilities

There are 3 ways for those residing in South Africa to receive medical card: public facilities which are free of charge for South African citizens, medical aid through private medical aid schemes or pay directly for their care.^23^ Seventeen percent of South African adults in 2017 were privately insured for their medical care.^23,24^ In terms of quality of life, differences between those attending private facilities and those attending public facilities aligned to the hypothesis: the means of 3 of the 4 quality-of-life subscales were higher in the group attending private facilities, with only spiritual well-being being higher in the public facility group. A difference in quality of life is commonly seen among different socioeconomic groups, and this was explored to see if facility classification affected the mean quality-of-life ratings. An acknowledged limitation of this study was that 79% of participants (majority) in this study attended a private facility for their breast cancer diagnosis and treatments.

#### Patients and survivors

In this study, all quality of life subscales recieved a higher mean in the survivor group than in the patient group. It is important to understand breast cancer patients’ and survivor’s lived experiences through all phases of their cancer continuum, with different challenges presented at different stages resulting in different quality of life.^15^

Quality of life is multifactorial, and although breast cancer diagnosis was the common denominator among this population, differences in economic background, employment, and education need to be considered when evaluating quality of life among these 2 groups, particularly in the context of South Africa. Participants were either undergoing treatment or a survivor at the time of data collection, which as expected demonstrated different quality-of-life ratings as the groups are at different points of the cancer continuum. It is important to acknowledge the changes in quality of life one may experience through the cancer continuum. The researchers note the discrepancies in group sizes for comparative purposes.

## Study Limitations

The researchers note the main limitation of this study to be the sample size obtained. The questionnaire was made available at private and public health care facilities in the region, as well as through an online platform; however, due to the Covid-19 pandemic, hard-copy participation was affected and owing to socioeconomic circumstances, some did not have access to participate online.

The limitations of this study include the following:

Access to the Internet, resource limitation, and technological knowledge may have affected potential participant’s ability to participate. To try to counter-act this limitation as well as for further inclusion, hard-copies of the questionnaire were offered to numerous hospitals and medical facilities to place in their receptions and cancer wards/areas. However, due to the Covid-19 pandemic, some participants felt uncomfortable with completing a hard-copy questionnaire.
The questionnaire was only in English, therefore limiting participation of non-English members of the population.
The questionnaire was self-reported and completed by the participants. No medical records were obtained; therefore, accuracy of the reported clinical data cannot be assumed.
The discrepancy in group sizes between the patients and survivors, and the private facility and public facility groups, is a limitation of the study. The researchers acknowledge that for a fair and more accurate comparison to be drawn, similar group sizes would be needed.
Although the questionnaire is considered a reliable tool (0.89; 0.93), it was not pilot tested on this sample and is therefore a limitation of this study.

Varying responses may have been affected by additional factors, such as current treatment phase and prescribed medications, stage of the disease, the Covid-19 pandemic, as well as current experience on the day of participation. Furthermore, the researchers acknowledge the differences and potential prejudice that may be present between those attending private facilities in comparison to public facilities. Investigating the differences in quality of life between patients attending private and public health care facilities provides the foundation for further research.

## Recommendations for Future Research

As research on cancer is continuing, as well as an increase in cancer survival rate, further research is required on the short- and long-term effects of the breast cancer continuum and how this affects the population’s quality of life. Insight into quality of life in this population may identify the nature of problems patients experience. This can then be addressed and improved, informing future patients on what to expect and how to manage their health, improving well-being among the population, evaluating quality of care, and researching optimal management of cancer.^8^ It is also noteworthy that the variances in socioeconomic status among the South African population can result in vast differences in quality of life. Therefore, it is strongly recommended that further studies be undertaken to explore the differences in quality of life between patients seeking treatment in private health care compared with public health care.

## Conclusions

One needs to consider the lifespan development affected by breast cancer and that challenges vary among patients and survivors – and may also vary for a particular person – therefore research on lifespan effects of breast cancer is needed. In previous studies, breast cancer patients and survivors have reported numerous positive and negative effects from their cancer diagnosis, treatments and survivroship.^7^ This study provided preliminary information regarding quality of life in a South African breast cancer population

## Supplemental Material

sj-doc-1-bcb-10.1177_11782234241282519 – Supplemental material for Quality of Life in Female Breast Cancer Patients and Survivors in a South African Municipality Supplemental material, sj-doc-1-bcb-10.1177_11782234241282519 for Quality of Life in Female Breast Cancer Patients and Survivors in a South African Municipality by Rebecca Wilkinson and Lynn Smith in Breast Cancer: Basic and Clinical Research

## Declarations

**Ethics approval and consent to participate:** Ethical clearance was granted from the Faculty of Health Sciences Research Ethics Committee at the University of Johannesburg (REC-1048-2021) before commencement of the study. Permission was sought and granted from relevant health care facilities and their practitioners for distribution of the questionnaire at their facility, as well as permission from the National Health Research Database. The information letter preceding both the online and hard-copy versions of the questionnaire provided the participants with the aim and processes of participating in the study and were required to provide consent before commencing participation. Every precaution was taken to protect the privacy and confidentiality of the participants. Prior to participation, the participants were presented with an information letter regarding the purpose of the study, aims and objectives, as well as details of participation, such as risks and benefits, rights of participants, and responsibilities of the researchers. The consent form had to be signed (hard-copies) and tick-boxes selected (online participation) prior to completing the questionnaire.

**Consent for publication:** All authors of the study consented to publication. Participants signed consent for publication of the study.

**Availability of data and materials:** This article was created from research which was part of a Master’s dissertation ‘Quality of life and physical activity levels in female breast cancer patients and survivors in Ekurhuleni, South Africa’ completed by author R Wilkinson and supervised by author Dr L Smith (https://ujcontent.uj.ac.za/esploro/outputs/9926508807691).
