# Supplementary material for: Quality of Life in Female Breast Cancer Patients and Survivors in a South African Municipality
Source: Breast Cancer (Auckl). 2024 Oct 8;18:11782234241282519. doi: 10.1177/11782234241282519 (PMC11465291; doi:10.1177/11782234241282519)
Supplement: sj-doc-1-bcb-10.1177_11782234241282519 – Supplemental material for Quality of Life in Female Breast Cancer Patients and Survivors in a South African Municipality [file sj-doc-1-bcb-10.1177_11782234241282519.doc]

**
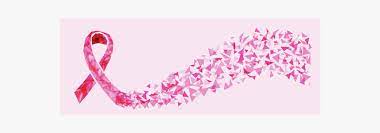
**

**QUALITY OF LIFE AND PHYSICAL ACTIVITY LEVELS IN FEMALE BREAST CANCER PATIENTS AND SURVIVORS IN EKUHURLENI, SOUTH AFRICA.**

**DEMOGRAPHIC INFORMATION / GENERAL INFORMATION:**

If there is a line, please write your answer. If there are circles, please select your answer.

1. What is your age / How old are you?

___________

1. At what age were you diagnosed with breast cancer / How old were you when you were diagnosed with breast cancer?

____________

1. In which year were you diagnosed with breast cancer / When were you diagnosed? (please give the year, i.e. 2003)

Year: ____________

1. What stage/level of breast cancer do you have / did you have when you were diagnosed? (mark the circle)

- Stage 1
- Stage 2
- Stage 3
- Stage 4
- I don't know the exact stage I have / had

1. What types of breast cancer do you / did you have? (mark the circle)

- Ductal / Lobular carcinoma in situ
- Invasive Ductal / Lobular carcinoma in situ
- Paget’s disease
- Inflammatory breast cancer
- Phyllodes Tumor
- Locally advanced breast cancer
- Metastatic breast cancer
- Other :...............................................................
- I am not sure of the exact type / I don’t know

1. Are you currently undergoing / having treatment? (Please mark the most appropriate answer)

- Yes
- No

1. What type / form of treatment are you receiving / getting or did you receive / get? (can mark multiple)
   - Surgery / operation
   - Chemotherapy
   - Radiation therapy
   - Hormone therapy
   - I decided not to have treatment / did not want treatment
   - Other:................................................
2. What sector/ type of facility are you / did you receive treatment from? (Please select the most appropriate answer)

- Private facility
- Public/government facility

1. Are you currently in remission/cancer-free? (Please select the most appropriate answer)

- Yes
- Still undergoing/having treatment
- No but no longer / not having treatment

1. If yes to previous question, how long have you been in remission/cancer free?

- Less than 1 year
- 1-5 years
- More than 5 years

1. Do you have any other health conditions (comorbidities)? (i.e. diabetes, high blood pressure, arthritis, etc.)

If yes please provide what conditions:.....................................................................................................

1. Which area of Ekurhuleni are you residing / living in? (circle the area you are living in)

- Nigel
- Olifantsfontein
- Springs
- Tembisa
- Tokoza
- Vosloorus
- Vorsterkroon
- Alberton
- Bedfordview
- Benoni
- Birchleigh
- Boksburg
- Brakpan
- Clayville
- Daveyton
- Dunnottar
- Edenvale
- Geduld
- Germiston
- Katlehong
- Kempton Park
- Kwa-themba
- Machenzieville

**GLOBAL PHYSICAL ACTIVITY QUESTIONNAIRE**

*World Health Organisation (2002)*

1. Does your work involve vigorous intensity (hard physical work) activity that causes large increase in heart rate or breathing for at least 10 minutes continuously / at a time? (an example of vigorous intensity exercise includes running, lifting and carrying heavy loads, digging, construction or swimming. These activities are often very tiring and physically straining). (mark the circle)

- Yes
- No (skip to question 16)
- I am unemployed (skip to question 19)
- I am a student (skip to question 19)

1. In a typical/normal week how many days do you do vigorous intensity activity as part of your work? (circle the number of days)

0 1 2 3 4 5 6 7

1. How much time do you spend doing vigorous intensity activity at work on a typical day? (please specify in minutes).

1. Does your work involve moderate intensity (some physical work) activity that causes small increases in breathing and heart rate for at least 10 minutes continuously / at a time? (an example of moderate intensity exercise includes walking and carrying light objects. These activities are not completely tiring or physically straining but do require effort to perform). (mark the circle)

- Yes
- No (skip to question 19)

1. In a typical / normal week how many days do you do moderate intensity activity as part of your work? (circle the number of days)

0 1 2 3 4 5 6 7

1. How much time do you spend doing moderate intensity activity at work on a typical day? (please specify in minutes).
2. Do you walk or use a bicycle for at least 10 minutes continuously / at a time to get to and from places? (mark the circle)

- Yes
- No (skip to question 22)

1. In a typical / normal week how many days do you walk or bicycle for at least 10 consecutive minutes to get to and from places? (circle the number of days)

0 1 2 3 4 5 6 7

1. How much time do you spend walking or bicycling for travel on a typical day? (please specify in minutes).
2. Do you do any vigorous intensity sports, fitness or recreational (leisure) (hard physical exercise) activities that cause large increases in breathing or heart rate (like running or football) for at least 10 minutes consecutively / at a time? (mark the circle)

- Yes
- No (skip to question 25)

1. In a typical / normal week how many days do you do vigorous intensity sports, fitness or recreation (leisure) activities? (circle the number of days)

0 1 2 3 4 5 6 7

1. How much time do you spend doing vigorous intensity sports, fitness or recreation (leisure) activity on a typical day? (please specify in minutes).
2. Do you do any moderate intensity sports, fitness or recreational (leisure) (somewhat hard physical exercise) activities that cause small increases in breathing or heart rate (like cycling, swimming or volleyball) for at least 10 consecutively / at a time? (mark the circle)

- Yes
- No (skip to question 28)

1. In a typical / normal week how many days do you do moderate intensity sports, fitness or recreation (leisure) activities? (circle the number of days)

0 1 2 3 4 5 6 7

1. How much time do you spend doing moderate intensity sports, fitness or recreation (leisure) activity on a typical day? (Please specify in minutes).
2. How much time do you spend sitting, reclining or laying back on a typical day? (mark the circle)

- 1-2 hours
- 3-4 hours
- 5-6 hours
- >7 hours

**QUALITY OF LIFE PATIENT/CANCER SURVIVOR VERSION**

*Ferrell, Hassey-Dow and Grant. (2012)*

Using a 0-10 scale, circle the number which most represents how you feel towards the question.

***Physical Well-being***

To what extent is the following a problem for you:

1. **Fatigue (feeling tired/drained/exhausted/run-down)**

0 1 2 3 4 5 6 7 8 9 10 No problem Severe / Big problem

1. **Appetite changes (changing in eating patterns/amounts/feeling hungry)**

0 1 2 3 4 5 6 7 8 9 10 No problem Severe / Big problem

1. **Aches and pains (soreness)**

0 1 2 3 4 5 6 7 8 9 10 No problem Severe / Big problem

1. **Sleep changes (decrease sleep time, unable to fall asleep, waking up tired, increased waking up during the night)**

0 1 2 3 4 5 6 7 8 9 10 No problem Severe / Big problem

1. **Constipation (unable to go to the bathroom for bowel/tummy movements)**

0 1 2 3 4 5 6 7 8 9 10 No problem Severe / Big problem

1. **Nausea (feeling unwell – stomach discomfort, dizzy and urge to vomit)**

0 1 2 3 4 5 6 7 8 9 10 No problem Severe / Big problem

1. **Menstrual (period) changes or fertility (ability to fall pregnant)**

0 1 2 3 4 5 6 7 8 9 10 No problem Severe / Big problem

1. **Rate your overall physical health (body health)**

0 1 2 3 4 5 6 7 8 9 10 Extremely poor /bad Excellent / Very good

***Psychological / Mental Well-being:***

1. How difficult / hard is it for you to **cope** **(deal with, overcome, manage)** today as a result of your disease and treatment?

0 1 2 3 4 5 6 7 8 9 10 Not at all difficult / hard Very difficult / hard

1. How good is your **quality of life (enjoyment of life, comfortable, healthy and able to participate in activities)**?

0 1 2 3 4 5 6 7 8 9 10 Extremely poor / bad Excellent / Very good

1. How much **happiness (joy, satisfaction, glad)** do you feel?

0 1 2 3 4 5 6 7 8 9 10 None at all / nothing A great deal / a lot

1. Do you feel like you are **in control** **(have power over)** of things in your life?

0 1 2 3 4 5 6 7 8 9 10 Not at all Completely / Yes

1. How **satisfying (fulfilled)** is your life?

0 1 2 3 4 5 6 7 8 9 10 Not at all Completely / Very

1. How is your present ability **to concentrate or to remember (focus when doing something and remembering things that have happened)** things?

0 1 2 3 4 5 6 7 8 9 10 Extremely poor / bad Excellent / Very good

1. How **useful (valuable, able to do what you need, feeling wanted)** do you feel?

0 1 2 3 4 5 6 7 8 9 10 Not at all Extremely / Very

1. Has your illness or treatment caused changes in your **appearance (how you look)**?

0 1 2 3 4 5 6 7 8 9 10 Not at all Extremely / A lot

1. Has your illness or treatment caused changes in your **self-concept** **(the way you see yourself)**?

0 1 2 3 4 5 6 7 8 9 10 Not at all Extremely / A lot

How distressing / upsetting / stressful is the following aspects of your illness and treatment

1. **Initial diagnosis (when you were first diagnosed)?**

0 1 2 3 4 5 6 7 8 9 10 Not at all distressing Very distressing

1. **Cancer treatments? (how you were treated for your cancer - chemo, radiation, surgery, hormone therapy, etc.)**

0 1 2 3 4 5 6 7 8 9 10 Not at all distressing Very distressing

1. **Time since my treatment** was completed **(since you stopped/finished your treatment)**?

0 1 2 3 4 5 6 7 8 9 10 Not at all distressing Very distressing

1. How much **anxiety** **(feeling tense, worried, concerned)** do you have?

0 1 2 3 4 5 6 7 8 9 10 None at all A great deal / A lot

1. How much **depression (feeling sad, hopeless, upset)** do you have?

0 1 2 3 4 5 6 7 8 9 10 None at all A great deal / a lot

To what extent are you fearful / scared of:

1. **Future diagnostic checks (your check-ups and retesting)**?

0 1 2 3 4 5 6 7 8 9 10 No fear / Not scared Extreme fear / Very scared

1. **A second cancer (getting cancer somewhere else in your body)**?

0 1 2 3 4 5 6 7 8 9 10 No fear / Not scared Extreme fear / Very scared

1. **Recurrence** **(your cancer coming back again)** of your cancer?

0 1 2 3 4 5 6 7 8 9 10 No fear / Not scared Extreme fear / Very scared fear

1. **Spreading (expanding/metastasis)** of your cancer?

0 1 2 3 4 5 6 7 8 9 10 No fear / Not scared Extreme fear / Very scared fear

***Social concerns***

1. How distressing **(worry, sad, concern)** has your illness been on your **family**?

0 1 2 3 4 5 6 7 8 9 10 Not at all A great deal / a lot

1. Is the amount of **support** **(comfort, encouragement, assistance, help)** you receive from others sufficient to meet your personal needs?

0 1 2 3 4 5 6 7 8 9 10 Not at all A great deal / a lot

1. Is your continuing healthcare interfering with your **personal relationships (your friendships, partnerships, relationships)**?

0 1 2 3 4 5 6 7 8 9 10 Not at all A great deal / a lot

1. Is your **sexuality** **(sexual feelings, thoughts and attractions)** impacted by your illness?

0 1 2 3 4 5 6 7 8 9 10 Not at all A great deal / a lot

1. To what degree has your illness and treatment interfered with your **employment (work)**?

0 1 2 3 4 5 6 7 8 9 10 No problem Severe / big problem

1. To what degree has your illness and treatment interfered with your **activities at home (cleaning, cooking, moving around)**?

0 1 2 3 4 5 6 7 8 9 10 No problem Severe / big problem

1. How much **isolation** **(alone, lonely, separated)** do you feel is caused by your illness and treatment?

0 1 2 3 4 5 6 7 8 9 10 None A great deal / a lot

1. How much **financial burden** **(debt, reduced money, having to save more now)** have you incurred / gained / got as a result of your illness and treatment?

0 1 2 3 4 5 6 7 8 9 10 None A great deal / a lot

***Spiritual Well-being***

1. How important to you is participation in **religious activities** **(i.e. praying, going to church, etc.)**

0 1 2 3 4 5 6 7 8 9 10 Not at all important Very important

1. How important to you are **spiritual (religious) activities** such as the ones mentioned above?

0 1 2 3 4 5 6 7 8 9 10 Not at all important Very important

1. How much has your **spiritual life (religious) changed** as a result of your cancer diagnosis?

0 1 2 3 4 5 6 7 8 9 10 Less important More important

1. How much **uncertainty** **(unsure, doubt, sceptical, mistrust)** do you feel about your future?

0 1 2 3 4 5 6 7 8 9 10 Not at all uncertain Very uncertain

1. To what extent has your illness made **positive (better, good) changes** in your life?

0 1 2 3 4 5 6 7 8 9 10 None at all A great deal / a lot

1. Do you sense **purpose or mission** **(feeling needed, useful, a reason to live)** for your life or a reason for being alive?

0 1 2 3 4 5 6 7 8 9 10 None at all A great deal / a lot

1. How **hopeful** **(trust, expectation, positive)** do you feel?

0 1 2 3 4 5 6 7 8 9 10 Not at all hopeful Very hopeful


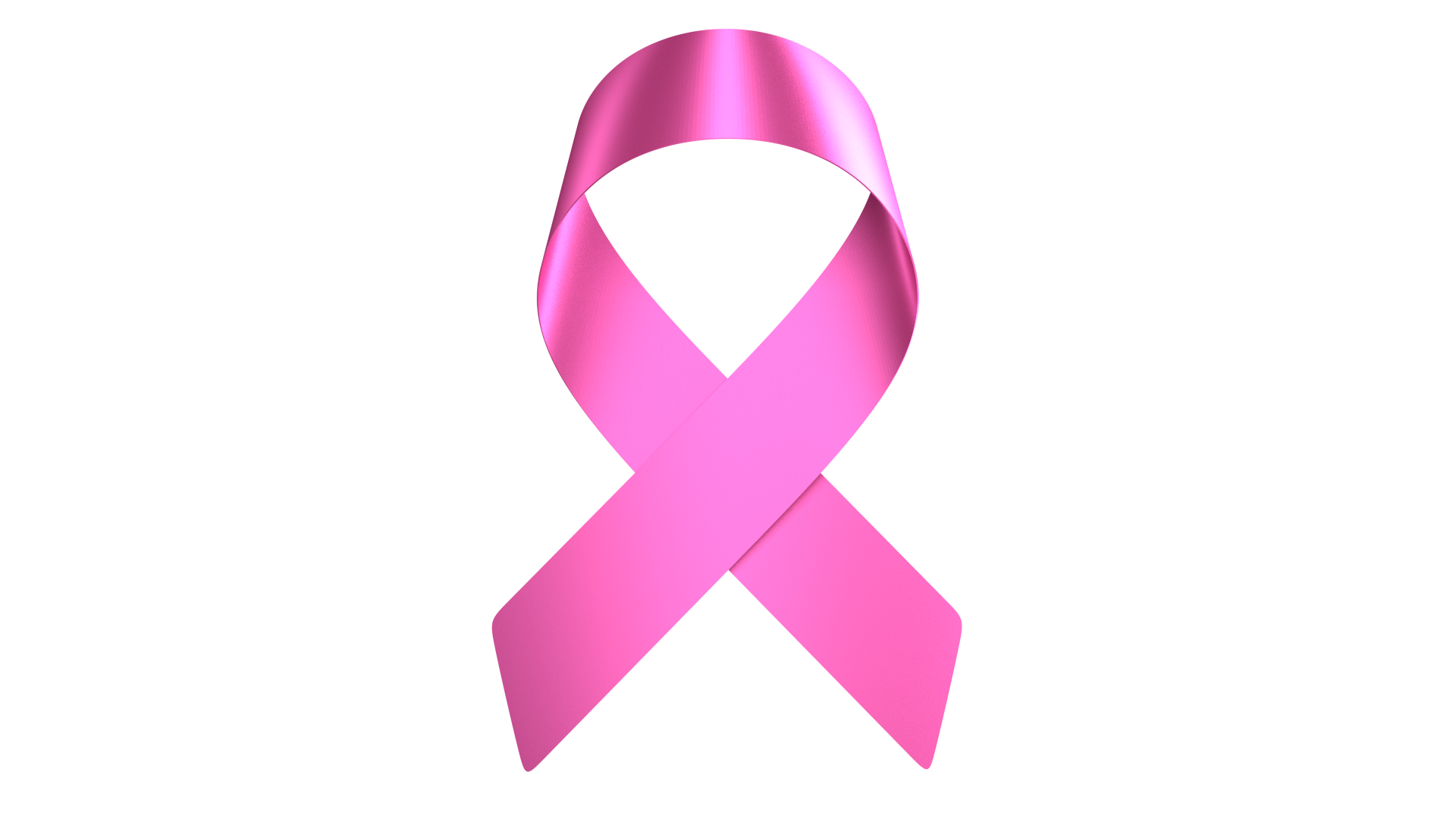


Thank you for your participation in this research!

Should any of the above questions have caused negative emotions (i.e. anxiety, depression, sadness or concern), we advise you discuss which elements affected you with your oncologist or your primary doctor who can then recommend coping strategies (in the form of therapy, medications, copy mechanisms or referral to another healthcare practitioner who specialises in your concern).
